# Reticulocalbin‐2 in the Hippocampus Improves Cognitive Function of Diabetic Mice

**DOI:** 10.1002/brb3.71336

**Published:** 2026-04-15

**Authors:** Ting Zhang, Simiao Tang, Jiahui Cheng, Biao Hu, Ling‐Qi Xie, Yalun Cheng, Xin Chen, Genqing Xie

**Affiliations:** ^1^ Jishou University School of Medicine Jishou Hunan China; ^2^ The First People's Hospital of Xiangtan City Xiangtan Hunan China; ^3^ Graduate Collaborative Training Base of The First People's Hospital of Xiangtan City, Hengyang Medical School University of South China Hengyang Hunan China; ^4^ Department of Endocrinology, Endocrinology Research Center Xiangya Hospital of Central South University Changsha Hunan China

**Keywords:** Diabetic cognitive dysfunction, GSK3β, reticulocalbin‐2, synapsin, Tau

## Abstract

**Purpose:**

To investigate the underlying mechanism linking diabetes to an increased risk of Alzheimer's disease (AD), specifically by examining the role of reticulocalbin‐2 (RCN2) in the hippocampus.

**Methods:**

Diabetes‐associated cognitive impairment was examined using db/db mice on a C57BL/6J background. Genetic and virus‐mediated approaches were employed to achieve hippocampal knockdown, conditional deletion, or overexpression of RCN2. Cognitive function and synaptic integrity were assessed using behavioral, molecular, and histological analyses. In addition, the GSK3β–Tau signaling pathway was analyzed to explore the molecular mechanisms underlying RCN2‐mediated synaptic regulation.

**Finding:**

This study reveals that decreased expression of RCN2 in the hippocampus is associated with cognitive impairment. Knockdown of hippocampal RCN2 directly led to cognitive decline and synaptic damage. Mechanistically, RCN2 functions by inhibiting the GSK3β‐Tau pathway, thereby delaying synaptic loss. Importantly, overexpression of RCN2 in the hippocampus was found to partially rescue cognitive decline, demonstrating its therapeutic potential for Alzheimer's disease in a diabetic mouse model.

**Conclusion:**

The findings suggest that RCN2 is a key neuroprotective protein, and targeting RCN2 could represent a promising therapeutic strategy for neurodegenerative diseases like Alzheimer's, particularly in the context of diabetes.

## Graphical abstract

Diabetes reduces hippocampal reticulocalbin‐2 (RCN2) expression, leading to increased GSK3β activity and Tau hyperphosphorylation, resulting in synaptic loss, neurofibrillary tangles, and cognitive decline associated with Alzheimer's pathology. We found that overexpression of RCN2 in the hippocampus partially rescued the cognitive decline and showed its treatment for Alzheimer's disease in diabetic mice.

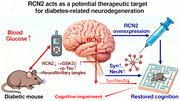

**Abbreviations**
AD: Alzheimer's disease
Ctrl: control
HG: high glucose
NG: normal glucose
NOR: Novel object recognition test
OE: overexpression
RCN2: reticulocalbin‐2
Syn: synapsin
WMW: Morris water maze
WT: wild‐type

## 1 Introduction

Patients with diabetes mellitus demonstrate a higher likelihood of progressing to Alzheimer's disease (AD) suggesting a possible link between the metabolic dysregulation seen in diabetes and the cognitive decline observed in AD (Stanciu et al. 2020; Wijesekara et al. 2018). Previous studies have revealed a potential relationship between diabetes and AD via oxidative stress (Sims‐Robinson et al. 2010), as individuals with type 2 diabetes exhibit an elevated risk of developing AD due to potential oxidative connections. Additionally, diabetes influences the progression of AD through its impact on glucose metabolism (Chatterjee and Mudher 2018), where dysregulation of central nervous system glucose levels contributes to decreased neuronal and synaptic function, as well as increased neuronal loss and its impact on glucose metabolism. Thus, identifying the key factors responsible for aggravating AD in diabetes could provide valuable insights into the prevention and treatment of both diabetes and AD.

The synapse, the junction between two neurons, plays a crucial role in cognitive function. It is the site where information is transmitted from one neuron to another, enabling various cognitive processes such as learning, memory, and decision‐making. Disruptions in synaptic function have been implicated in numerous neurological disorders, including AD (Wang et al. 2015). Therefore, investigating the role of synapses in cognition is essential for unraveling the complexities of cognitive dysfunction and developing potential therapeutic strategies.

Reticulocalbin‐2 (RCN2), a calcium‐binding protein primarily localized in the endoplasmic reticulum, has recently emerged as a potential player in lipolysis and many other disorders (Peng et al. 2022; Li et al. 2022). RCN2 has been implicated in regulating calcium homeostasis (Yao et al. 2023) and protein folding (Honore 2009), all of which are critical for proper cognitive function. It was reported that spinophilin, a protein that regulates synaptic transmission by targeting PP1 to the postsynaptic density, has been shown to interact with RCN2 (Di Sebastiano et al. 2016). Furthermore, conditional deletion of neurexin‐2, a protein that interacts with reticulocalbin‐2 (Lin et al. 2023), could lead to spontaneous seizures in mice (Haile et al. 2023). Considering the significant role of RCN2 in both neural and metabolic aspects, we wondered whether RCN2 was a contributor to AD progression during diabetes and could act as a novel therapeutic intervention for neurodegenerative diseases.

In this study, we demonstrated that deleting reticulocalbin‐2 in the hippocampus of mouse models leads to a decline in cognitive function, while overexpressing reticulocalbin‐2 in the hippocampus can improve cognitive function in diabetic mice. Additionally, RCN2 expression is negatively correlated with GSK3β and Tau. These findings suggest that RCN2 may serve as a pathological mechanism for cognitive impairment in diabetes and a potential target for the treatment of diabetic dementia.

## 2 Methods

### 2.1 Animal Models

C57BL/6J mice were acquired from Hunan SJA Laboratory Animal Company (Hunan, China).For diabetes models, db/db mice (age 8 weeks at the start of experiments, *n* = 5 per group) were used. RCN2‐floxed mice were created through the integration of dual flox sequences (ATAACTTCGTATAGCATACATTATACGAAGTTAT) at both exon 2 termini of RCN2 via Cyagen Biosciences' CRISPR/Cas9 technology (China). Camk II‐Cre mice were procured from Jackson Laboratory (USA). All experimental mice were of the C57BL/6J genetic background. These experimental animals were maintained under specific pathogen‐free conditions at the Laboratory Animal Research Center of Central South University, where ambient temperature was maintained at 22–24°C under a 12‐h light/dark cycle (lights on from 7:00 a.m. to 7:00 p.m.). The mice were provided ad libitum with a standard rodent diet (supplied by Hunan SJA Laboratory Animal Company, China) and tap water. Only healthy male mice were included in the study, with littermates serving as controls.

All animal care protocols and experiments were reviewed and approved by the Animal Care and Use Committees of the Laboratory Animal Research Center at Xiangya Medical School of Central South University.

### 2.2 Stereotaxic Surgery

Following confirmation of reflex absence, mice were profoundly anesthetized via intraperitoneal injection and secured in a stereotaxic apparatus. Intracerebral administrations were performed with a Hamilton syringe; the hippocampal injection coordinates were −2.1 mm anteroposterior (AP); −/+ 1.5 mm medio‐lateral (ML) relative to Bregma, and −1.8 mm dorso‐ventral (DV) from the dural surface. A 0.5 µL aliquot was infused into the hippocampal region at a constant rate of 0.05 µL/min. To prevent backflow, the needle was gradually withdrawn over 10 min postinjection. Surgical recovery was facilitated by maintaining animals on heating pads with continuous monitoring until full anesthesia recovery. The animals were kept in separate cages with unrestricted availability of standard diet and drinking water.

### 2.3 Morris Water Maze Test (MWM)

The cognitive performance of mice was evaluated using the MWM test, conducted according to standard procedures (Vorhees and Williams 2006). The Smart v3.0 tracking system quantified multiple behavioral parameters, including escape latency, total distance traveled, swimming velocity, and movement trajectories. During the training phase, all mice received four daily trials with a 60‐s cutoff period, repeated over five consecutive days with 15‐min rest intervals between trials. Animals failing to locate the platform within the allotted time were gently guided to the target and maintained there for 10 s. Following the 5‐day acquisition period, spatial memory retention was assessed through a probe trial. The time spent in the target quadrant was measured over 60 s, as well as the time to reach the platform along with the number of times the mice crossed the target location.

### 2.4 Novel Object Recognition Test (NOR)

The NOR tests were carried out as previously described (Jessberger et al. 2009). Following a 15‐min habituation period in an empty white arena, mice were reintroduced after 24 h to the same chamber containing two identical corner‐positioned objects and permitted free exploration until accumulating 30 s of investigative contact with both objects. Following a 24‐h interval, an object recognition assessment was conducted by introducing a novel object with counterbalanced placement in the chamber; rodent exploratory activity was videotaped for 6 min using a TV/VCR system. Exploration duration for individual objects was measured utilizing Corel VideoStudio Pro X5. The novel object recognition test results are presented as the percentage of exploration time devoted to the novel object/location relative to the total time spent investigating both objects.

### 2.5 Cell Culture

Cell lines of HT22 were obtained from the Type Culture Collection of the Chinese Academy of Sciences (Shanghai, China). HT22 cells were cultured in Dulbecco's modified Eagle medium (DMEM, Gibco C11965500BT, CA, USA) supplemented with 10% fetal bovine serum (Gibco 10099‐141, CA, USA) and 1% penicillin/streptomycin (10,000 units/mL penicillin and 10,000 µg/mL streptomycin; Gibco). Cells were maintained at a constant temperature of 37°C in a 5% CO_2_‐humidified incubator. HT22 cells were categorized into two treatment groups: normal glucose (25 mM glucose) and high glucose (55 mM glucose).

HT22 cells were transfected with RCN2 plasmids using Lipofectamine 2000 reagent following the manufacturer's instructions (Invitrogen), and the cells were transfected with RCN2 siRNA (supplied by Gene Pharma, Shanghai, China) using Lipofectamine RNAiMAX Transfection Reagent (Thermofisher, CA, USA) according to the manufacturer's protocol.

### 2.6 Immunofluorescence (IF)

Transfected/treated cells or murine brain sections were immobilized and maintained for 24–48 h at 4°C with the following primary antibodies: anti‐RCN2 (10193‐2‐AP, Proteintech, 1:2000), anti‐Synaptophysin (AB1543, Sigma‐Aldrich, 1:1000), and anti‐NeuN (ab177487, Abcam, 1:500). Subsequently, the samples were exposed for 1 h at 37°C to Alexa Fluor 568‐, Alexa Fluor 488‐, or Alexa Fluor 647‐conjugated secondary antibodies (Invitrogen). Nuclear staining was conducted with DAPI (1 µg/ml) (Sigma). Image acquisition was conducted with a confocal microscope (Olympus FV1000). For fluorescence quantification, multichannel images were separated into individual channels and transformed into 8‐bit grayscale, followed by duplication to generate binary images. Subsequently, a region of interest surrounding the target was delineated and emphasized. Background signals were eliminated using a rolling ball algorithm to produce a binary image with two pixel intensities: black = 0 and white = 255. Following measurement parameter configuration and “Redirect to” line assignment to the duplicated image name, fluorescence intensities within the binary images were examined.

### 2.7 Real‐Time PCR

RNA extraction from cellular and tissue samples was carried out using Trizol reagent. Following cDNA synthesis with SuperScript III reverse transcriptase, quantitative PCR amplification was conducted on the ABI 7500‐Fast Real‐Time PCR System. Gene‐specific primers and the TaqMan Universal Master Mix Kit were custom‐designed and acquired from Taqman. All experimental reagents and kits were obtained from Life Technologies. Gene expression levels were comparatively analyzed employing the 2−ΔΔCt calculation method. Technical replicates included a minimum of duplicate wells per sample.

### 2.8 Western Blotting

Following ice‐cold PBS washes, cellular lysis was carried out in RIPA buffer (20 mM Tris‐HCl, pH 7.5, 1 mM EDTA, 1 mM EGTA, 150 mM NaCl, 2.5 mM sodium pyrophosphate, 1% NP‐40, 1% sodium deoxycholate, 1 mM Na_3_VO_4_, and 1 mM β‐glycerophosphate) supplemented with protease inhibitor cocktail for 20 min on ice. Following centrifugation at 14,000 rpm for 20 min at 4°C, the supernatant was harvested. Protein concentration was determined using a BCA Protein Assay Kit (Pierce), according to the manufacturer's instructions, with bovine serum albumin as the standard. Subsequently, the protein lysate was adjusted to a concentration of 5 mg/ml. Following electrophoresis, specimens were immunoprobed overnight at 4°C using manufacturer‐specified antibody concentrations, subsequent to which immunoblot signals were developed. Primary antibodies were used as follows: PHF (ab184951, 1:1000, Abcam), Syn (AB1543, 1:1000, Sigma‐Aldrich), GSK3β (12456T, 1:1,000; Cell Signaling Technology), RCN2 (10193‐2‐AP, 1:2000, Proteintech), and β‐actin (MABT825, 1:500, Sigma‐Aldrich).

## 3 Results

### 3.1 Diabetic Mice With Cognitive Impairment Showed Decreased RCN2 in the Hippocampus

**FIGURE 1:**
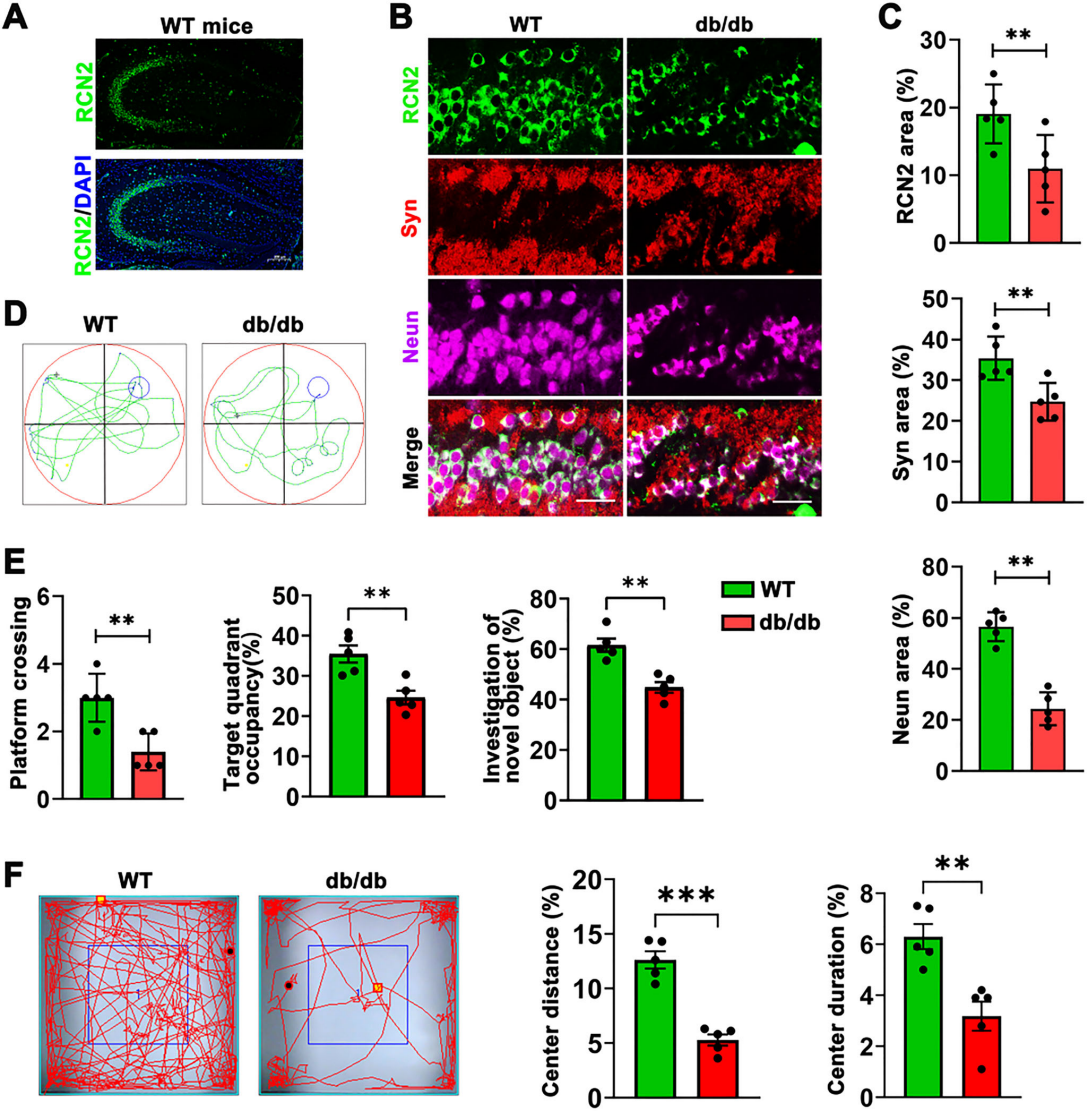
**Diabetes inhibits the expression of RCN2 and causes cognition impairment**. **(A)** Representative immunofluorescence images of hippocampus *RCN2* (green) staining in wild‐type mice. Scale bar, 200 µm. **(B)** Representative immunofluorescence images of *RCN2* (green), *Syn* (red), and *NeuN* (magenta) in the hippocampus of wild‐type and db/db mice (*n* = 5 per group). Scale bar, 50 µm, **(C)** Quantitation of the percentage area positive for *RCN2* (green), *Syn* (red), and *NeuN* (magenta) fluorescence in the hippocampus of WT and db/db mice, **(D)** Representative swimming paths of wild‐type (WT) control mice and db/db mice in the Morris water maze test, **(E)** Cognitive performance of WT and db/db mice in the Morris water maze test and novel object recognition tests (*n* = 5 per group). Parameters assessed include the number of platform crossings, the percentage of time spent in the target quadrant, and the exploration ratio of novel objects to familiar objects, and **(F)** Analysis of locomotor activity in the Open Field Test (OFT) for WT and db/db mice (*n* = 5). Bar graphs show the percentage of total distance traveled in the center area (left) and the percentage of time spent in the center area (right). Data are presented as mean ± s.e.m. The student's two‐sample *t*‐test was used to detect differences between the two groups. ***p < 0.001, **p < 0.05, and *p < 0.01.

To better investigate the relationship between cognition and diabetes, we first established a mouse model of cognitive impairment in diabetes. Specifically, db/db mice were continuously fed a high‐fat diet for 8 weeks. To explore whether the cognitive changes in diabetic mice are related to RCN2, RCN2 staining was performed on the hippocampus and showed there was a lower expression level of RCN2 in contrast to the control group (Figures 1A–1C). Additionally, immunofluorescence of hippocampus was conducted and the results showed the expression levels of the neuronal marker *NeuN* and synaptic protein marker synapsin (*Syn*) in the hippocampal region of diabetic mice were significantly lower compared to the control group (Figures 1B–1C), which further verified the cognitive decline in these mouse models.

Subsequently, a battery of behavioral assessments, comprising the Morris water maze, novel object recognition test, and open field test, was conducted to evaluate cognitive and behavioral performance in diabetic mice. Compared to normal mice, diabetic mice spent less time wandering in the target quadrant, made fewer platform crossings, and exhibited a significant reduction in activity in the center area, indicating impaired cognitive function and altered behavioral patterns in these mice (Figures 1D–1F). Consistent with the above results, these mice also showed significantly worse recognition ability in the novel object recognition test, as indicated by the investigation ratio of the novel object (Figure 1E).

Together, our results indicated that RCN2 decreased in the hippocampus in diabetic mice with cognitive impairment.

### 3.2 RCN2 Knockdown in Hippocampus Resulted in Cognitive Impairment

**FIGURE 2:**
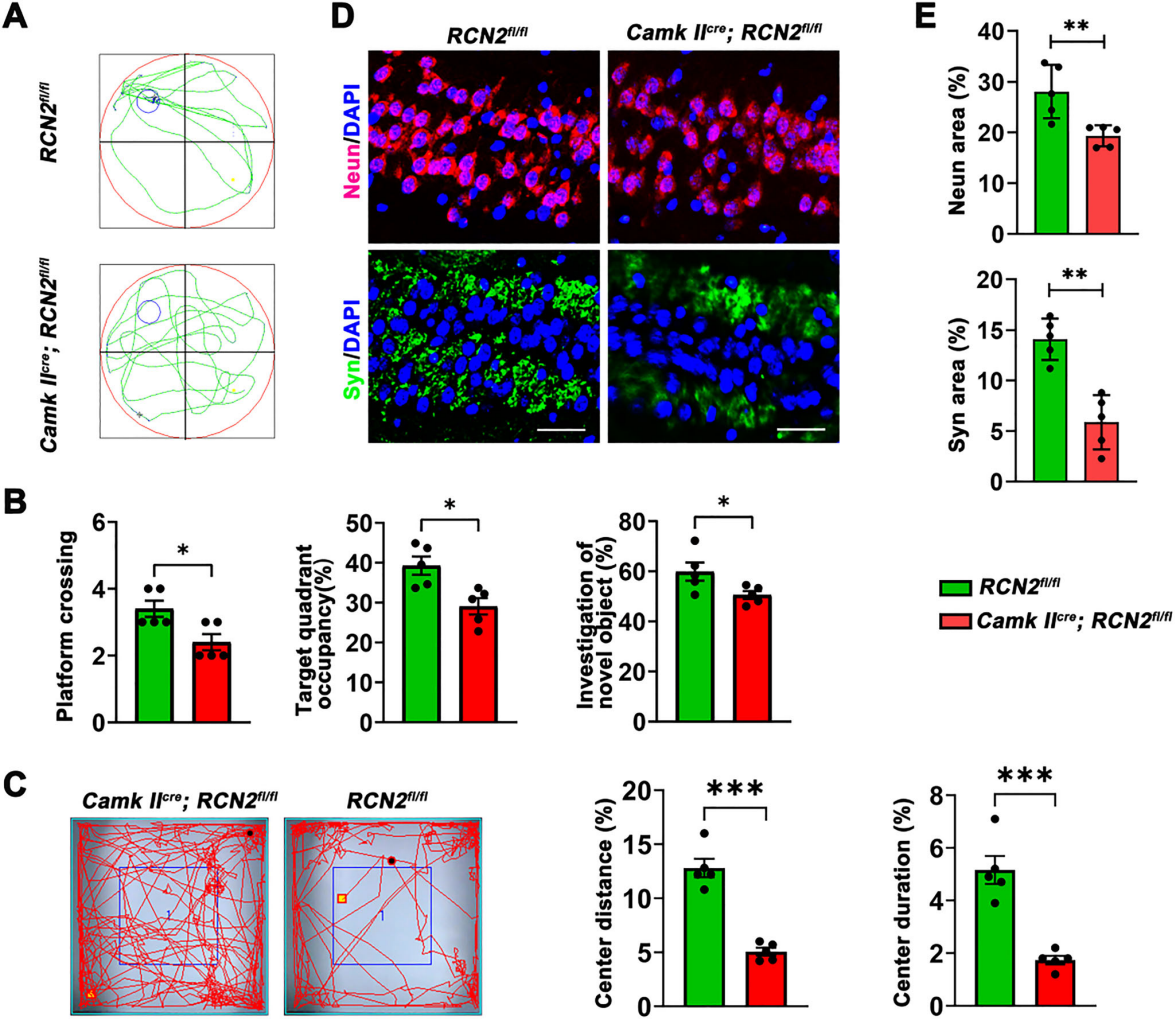
**Hippocampus RCN2 knockdown mice exhibit impaired cognitive function. (A)** Representative swimming paths of the control group and hippocampal‐specific RCN2 knockout mice in the Morris water maze test, **(B)** Cognitive performance of control and hippocampal‐specific RCN2 knockout mice in the Morris water maze and novel object recognition tests (*n* = 5). Parameters assessed include the number of platform crossings, the percentage of time spent in the target quadrant, and the ratio of investigation between novel and familiar objects, **(C)** Analysis of locomotor activity in the OFT for control and hippocampal‐specific RCN2 knockout mice (*n* = 5). Bar graphs show the percentage of total distance traveled in the center area (left) and the percentage of time spent in the center area (right), **(D)** Representative immunofluorescence images showing *NeuN* (red) and *Syn* (green) expression in the hippocampus of control and hippocampal‐specific RCN2 knockout mice (*n* = 5). Scale bar, 50 µm, and **(E)** Quantitation of the percentage area positive for *NeuN* (red) and *Syn* (green) in the hippocampus of different mouse groups. Data are presented as mean ± s.e.m. The student's two‐sample *t*‐test were used to detect differences between the two groups. ***p < 0.001,**p < 0.05, and *p < 0.01.

To further elucidate the involvement of RCN2 in diabetes‐associated cognitive dysfunction, RCN2‐floxed mice (RCN2 fl/fl mice) were bred with CamkII‐Cre mice to establish hippocampal‐specific RCN2 knockout animals (RCN2‐CamkII‐CKO). Camk II‐CreRCN2^fl/+^, RCN2^fl/fl^, or RCN2^fl/+^ was used as control. Similarly, we found that RCN2 knockout mice showed declined cognitive function compared to the control group. Specifically, in the acquisition process of the Morris water maze, the retention time spent in the target quadrant and times of platform crossings were significantly lower in the RCN2 knockout group than in the control group, as was the investigation ratio of novel objects from the novel object recognition test (Figures 2A–2B). Moreover, in the open field test, both the percentage of total distance traveled and the percentage of time spent in the center area were significantly reduced in the RCN2 knockout group compared with the control group (Figure 2C). In addition, immunofluorescence of the hippocampus also showed that there were reduced *Syn* and *NeuN* in the hippocampus‐specific RCN2 knockout group (Figures 2D–2E). These results strongly suggested that RCN2 played a crucial role in maintaining cognitive function.

### 3.3 Decreased Synaptic Count in Neuronal Cells After RCN2 Knockdown

**FIGURE 3:**
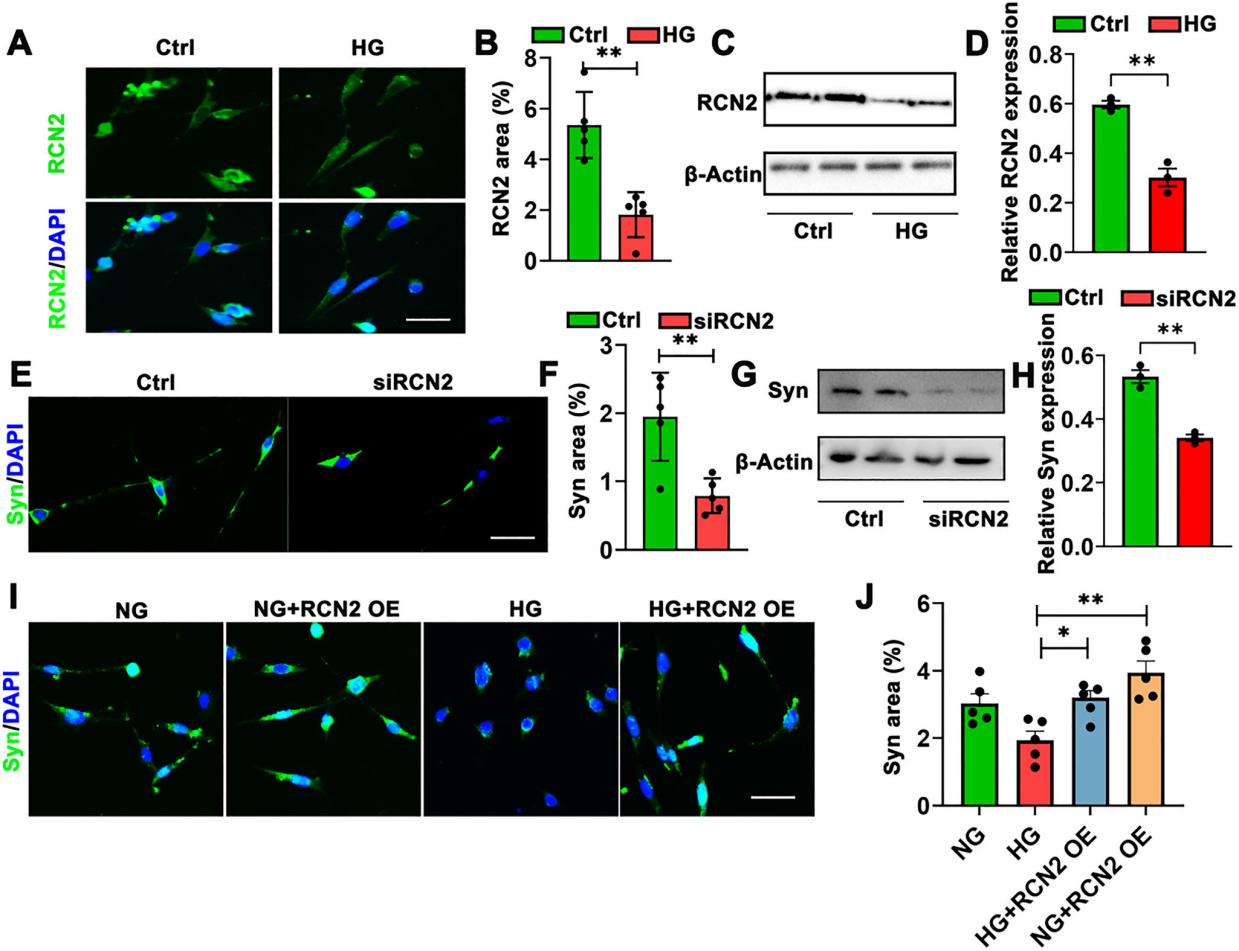
***RCN2* knockdown in HT22 led to loss of synapses. (A)** Representative immunofluorescence images (*n* = 5) showing RCN2 (green) expression in the HT22 cell line treated with normal glucose (Ctrl) or high glucose (HG) (*n* = 5 per group). Scale bar, 50 µm, **(B)** Quantitation of the percentage area positive for *RCN2* (green) in Ctrl and HG groups, **(C)** Representative western blot analysis of RCN2 protein levels in HT22 cells under Ctrl and HG conditions (*n* = 3 per group), **(D)** Quantification of RCN2 expression normalized to loading control (β‐Actin) in Ctrl and HG groups, **(E)** Representative immunofluorescence images showing *Syn* (green) expression in HT22 cells transfected with control siRNA (Ctrl) or RCN2 siRNA (*siRCN2*) (*n* = 5 per group). Scale bar, 50 µm, **(F)** Quantitation of the percentage area positive for *Syn* (green) fluorescence in Ctrl and siRCN2 group, **(G)** Representative Western blot analysis of Syn protein levels in HT22 cells following Ctrl or *siRCN2* treatment (*n* = 3 per group), **(H)** Quantification of Syn expression normalized to loading control (β‐Actin) in Ctrl and *siRCN2* groups, **(I)** Representative immunofluorescence images showing *Syn* (green) expression in HT22 cells under normal glucose (NG), high glucose (HG), HG with RCN2 overexpression (HG + RCN2 OE), and NG with RCN2 overexpression (NG + RCN2 OE) conditions (*n* = 5 per group). Scale bar, 50 µm, **(J)** Quantitation of the percentage area positive for *Syn* (green) fluorescence in NG, HG, HG + RCN2 OE, and NG + RCN2 OE groups. Data are presented as mean ± s.e.m. Statistical comparisons between two groups were performed using a two‐tailed Student's *t*‐test, while comparisons among four groups were analyzed using the Kruskal–Wallis test. ***p < 0.001, **p < 0.05, and *p < 0.01.

To figure out if RCN2 was involved in cognitive impairment of diabetic mice, we treated mouse hippocampal neuronal cell line HT22 with DMEM with high glucose (HG) or normal glucose (NG). Immunofluorescence analysis revealed a significant reduction in RCN2 expression levels under high glucose conditions (Figures 3A–3D).

To further elucidate the impact of RCN2 on synapses, we transfected HT22 with *RCN2 siRNA*. The results showed that HT22 transfected with *SiRCN2* exhibited a lower level of RCN2 (Figures 3E–3H). Besides, the fluorescence density of Syn also significantly decreased, indicating RCN2 affected synaptic connections in the hippocampus. Next, we overexpressed *RCN2* in HT22 cells. As expected, RCN2‐overexpressed HT22 cells showed higher expression of Syn and restored the decline induced by high glucose intervention (Figures 3I–3J). Therefore, our data revealed that RCN2 played a role in synaptic loss in the hippocampus.

### 3.4 RCN2 Delays Synaptic Loss Induced by High Sugar Through the GSK3β‐Tau Pathway

**FIGURE 4:**
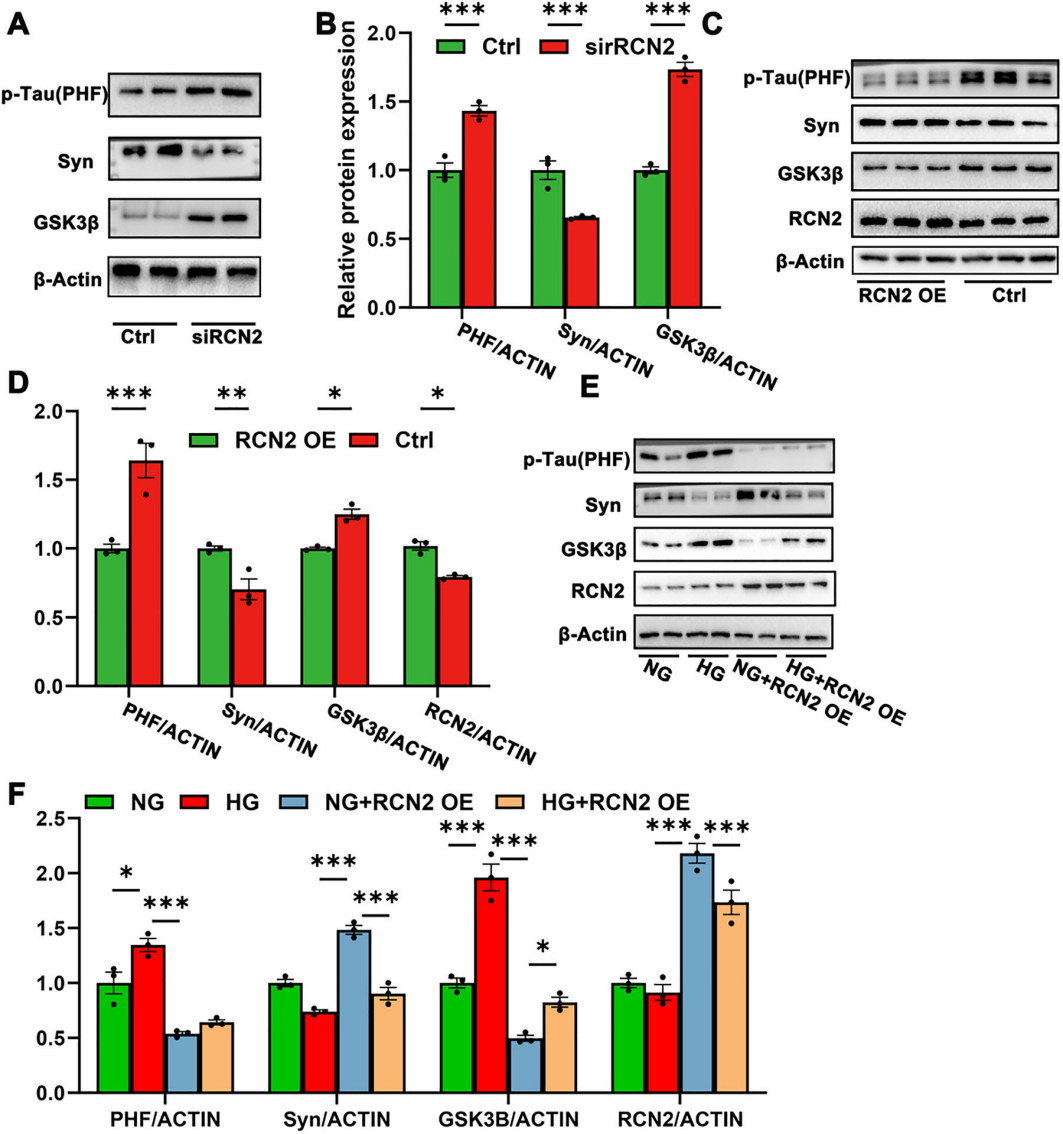
**Synaptic loss induced by high glucose was subdued by RCN2 via the GSK3β‐Tau pathway. (A)** Representative western blot analysis of p‐Tau (PHF), Syn, and GSK3β protein levels in HT22 cells treated with control siRNA (Ctrl) or RCN2 siRNA (siRCN2) (*n* = 3 per group), **(B)** Quantification of p‐Tau (PHF), Syn, and GSK3β expression normalized to loading control (β‐Actin) in Ctrl and siRCN2 groups, **(C)** Representative western blot analysis of p‐Tau (PHF), Syn, GSK3β, and RCN2 protein levels in HT22 cells overexpressing RCN2 (RCN2 OE) or control (Ctrl) (*n* = 3 per group), **(D)** Quantification of p‐Tau (PHF), Syn, GSK3β, and RCN2 expression normalized to loading control (β‐Actin) in Ctrl and RCN2 OE groups, **(E)** Representative western blot analysis of p‐Tau (PHF), Syn, and GSK3β, and RCN2 in HT22 cells under normal glucose (NG), high glucose (HG), HG with RCN2 overexpression (HG + RCN2 OE), and NG with RCN2 overexpression (NG + RCN2 OE) conditions (*n* = 3 per group), and **(F)** Quantification of p‐Tau (PHF), Syn, GSK3β, and RCN2 expression normalized to loading control (β‐Actin) in NG, HG, HG + RCN2 OE, and NG + RCN2 OE groups. Data are presented as mean ± s.e.m. Statistical comparisons between two groups were performed using a two‐tailed Student's *t*‐test, while comparisons among four groups were analyzed using the Kruskal–Wallis test. ***p < 0.001, **p < 0.05, and *p < 0.01.

The GSK3β‐Tau pathway has been a focus of research in the context of AD and synaptic function. GSK3β is known to phosphorylate tau protein, leading to its abnormal hyperphosphorylation and aggregation, which can contribute to synaptic dysfunction and cognitive impairment in AD (Toral‐Rios et al. 2020; Amaral et al. 2021). Inhibiting GSK3β has demonstrated improvements in synaptic and cognitive functions in AD‐like mouse models (Zhou et al. 2022). We then investigated if RCN2 modulated this pathway. We found that knockdown of RCN2 in HT22 cells results in decreased Syn expression and increased levels of phosphorylated Tau and GSK3β (Figures 4A–4B). On the contrary, RCN2‐overexpressed HT22 cells exhibited decreased phosphorylated tau and GSK3β (Figures 4C–4D). Moreover, RCN2 overexpression in HT22 treated with HG led to improved Syn expression and lower levels of Tau and GSK3β (Figures 4E–4F). Thus, we speculated that RCN2 inhibited GSK3β‐Tau signaling to delay synaptic loss.

### 3.5 Overexpression of RCN2 in Hippocampus Rescued Cognitive Decline in Diabetic Mice

**FIGURE 5:**
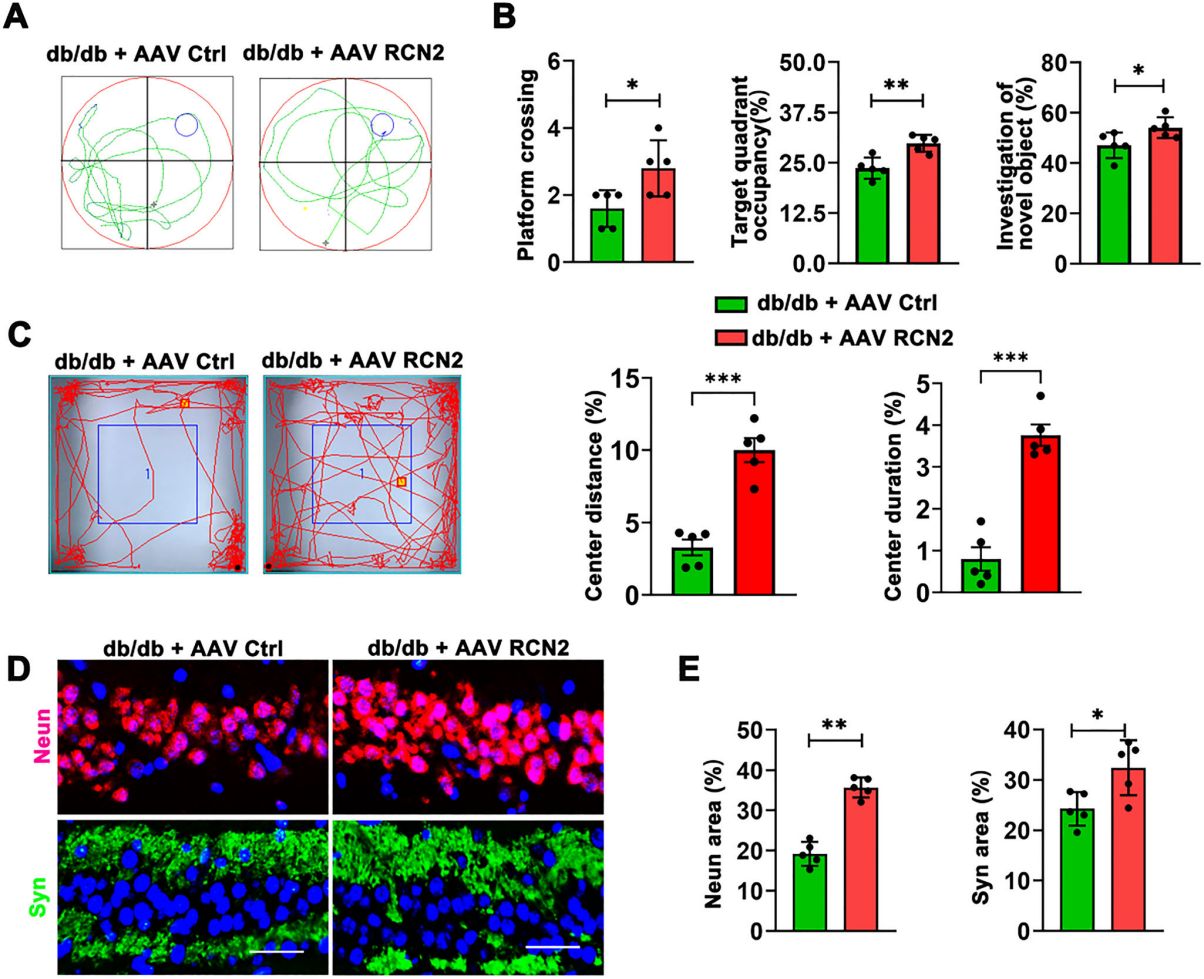
**Diabetes‐induced cognitive decline can be reversed by overexpression of RCN2 in the hippocampus. (A)** Representative swimming paths of db/db control (db/db + AAV Ctrl) and db/db mice with hippocampal‐specific RCN2 overexpression (db/db + AAV RCN2) in the Morris water maze test, **(B)** Cognitive performance of db/db + AAV Ctrl and db/db + AAV RCN2 mice in the Morris water maze and novel object recognition tests (*n* = 5 per group). Parameters assessed include the number of platform crossings, the percentage of time spent in the target quadrant, and the investigation ratio of the novel object to the familiar object, **(C)** Analysis of locomotor activity in the Open Field Test (OFT) for db/db + AAV Ctrl and db/db + AAV RCN2 mice (*n* = 5). Bar graphs show the percentage of total distance traveled in the center area and the percentage of time spent in the center area, **(D)** Representative immunofluorescence images showing *NeuN* (red) and *Syn* (green) expression in the hippocampus of db/db + AAV Ctrl and db/db+AAV RCN2 mice (*n* = 5). Scale bar, 50 µm, and **(E)** Quantitation of the percentage area positive for *NeuN* (red) and *Syn* (green) fluorescence in the hippocampus of db/db + AAV Ctrl and db/db + AAV RCN2 mice. Data are presented as mean ± s.e.m. The student's two‐sample *t*‐test were used to detect differences between the two groups. ***p < 0.001, **p < 0.05, and *p < 0.01.

As shown above, depletion of RCN2 showed serious impairment for cognitive function; we then wondered if increased RCN2 could benefit cognition in diabetic mice. Thus, AAV‐RCN2 was administered into the hippocampal region of diabetic mice exhibiting cognitive impairment, thereby inducing hippocampus‐specific overexpression of RCN2. With 20 week‐aged mice, we evaluated cognitive function through the above behavioral experiments and found RCN2‐overexpressing mice showed significant improvement in the water maze test as indicated by shorter time to reach the central platform, increased target quadrant dwell time, and platform crossing frequency relative to controls (Figures 5A–5B). In addition, RCN2‐overexpressed mice also exhibited improved recognition ability in the novel object recognition experiment, along with significantly increased percentages of both distance traveled and time spent in the center area during the open field test (Figure 5C). Consistently, immunohistochemical analysis revealed increased fluorescence intensity of *Syn* and *NeuN* in the hippocampus of RCN2‐overexpressing mice relative to controls (Figures 5D–5E). Together, our findings revealed RCN2 in the hippocampus was sufficient to rescue cognitive decline in diabetic mice.

## 4 Discussion

Current research on the relationship between diabetes and AD has uncovered several mechanisms linking the two conditions. These include the impact of hyperglycemia and hypoglycemia on neuronal and synaptic function (Chatterjee and Mudher 2018; Wei et al. 2021; Hamzé et al. 2022), the shared amyloid aggregation and deposition, as well as the bidirectional association between type 2 diabetes and AD. Hamze and Nguyen et al. demonstrated that prediabetic or type 2 diabetic individuals exhibit an elevated risk for Alzheimer's disease and other dementia subtypes (2022; 2020). Insulin resistance is one mechanism through which diabetes can contribute to dementia (Candasamy et al. 2020; Kellar and Craft 2020). Both diabetes mellitus and Alzheimer's disease represent multifactorial disorders with diverse biological pathways, prompting the proposal of terms like ‘diabetes type 3’ or ‘brain diabetes’ to comprehensively describe their potential shared pathogenic mechanisms (Michailidis et al. 2022). However, the research also highlights current shortcomings and limitations, emphasizing the need for further studies to fully elucidate the link between diabetes and AD. The overlap and intersection of molecular pathways, such as oxidative stress (Sims‐Robinson et al. 2010; González et al. 2022), the formation of advanced glycation end products, and impairments in central nervous system insulin signaling, require more comprehensive understanding. Additionally, while the association between diabetes and AD is well‐established, more research is needed to develop effective therapeutic targets and interventions to address the shared pathological traits and mechanisms between the two conditions.

As a debilitating neurodegenerative disorder, AD is characterized by the accumulation of abnormal proteins (Wu et al. 2021), synaptic dysfunction (Tonnies and Trushina 2017; Meftah and Gan 2023), and cognitive decline. Synapses are vital structures for neuronal communication, and their dysfunction is a core feature of AD. Synapsin, a presynaptic terminal‐enriched phosphoprotein, is critically involved in modulating neurotransmitter release and synaptic formation (Hilfiker et al. 1999; Cesca et al. 2010). Disruptions in synapsin expression and function have been implicated in AD pathogenesis (Qin et al. 2004). Elevated levels of synapsin in cerebrospinal fluid have been detected in early AD, suggesting a possible compensatory response to synaptic impairment (Perrin et al. 2011). Conversely, reductions in synapsin levels have been observed in later stages of the disease, correlating with cognitive decline (Ng et al. 2022). Research on the role of synapses in AD has revealed that synapse damage and loss are fundamental to the pathophysiology of AD, leading to reduced cognitive function (Colom‐Cadena et al. 2020). Studies have also shown that the GSK3β‐Tau pathway may play a vital role in synaptic dysfunction and cognitive impairment in AD (Jackson et al. 2019; Griffiths and Grant 2023). While there is extensive research on the relationship between diabetes and AD, including the potential biological mechanisms linking the two conditions, such as the impact of hyperglycemia and hypoglycemia on neuronal and synaptic function, the specific relationship between diabetes and synapses in the context of AD is an area that requires further investigation (Sims‐Robinson et al. 2010; Hamze et al. 2022).

Recent investigations into RCN2 have uncovered its diverse roles across various biological processes and diseases. In the realm of cancer biology, a study exploring nasopharyngeal carcinoma progression revealed that RCN2 plays a crucial role by regulating calcium flow and mitochondrial apoptosis, and the study demonstrated that the knockout of RCN2 effectively inhibited the malignant biological behavior of tumor cells (Yao et al. 2023; Wang et al. 2017; Ning et al. 2023). Furthermore, RCN2 has been implicated in bone marrow function, where it facilitates fat lipolysis to regulate both osteogenesis and lymphopoiesis, suggesting its potential importance in bone marrow function and immune responses (Gugala 2022). Additionally, research has identified RCN2 as a partial inhibitor of calcineurin activity, providing valuable insights into calcineurin regulation and offering potential avenues for therapeutic strategies (Wardaszka et al. 2021; Miyazaki et al. 2011). In the context of cardiovascular disease, investigations into RCN2 as a potential biomarker and therapeutic target for atherosclerosis underscore its association with this condition (Li et al. 2022; Li et al. 2019; Chang et al. 2019). Based on our research team's previous study, RCN2 may act as a mechanosensitive lipolytic factor, with bone marrow macrophages secreting RCN2 abundantly following mechanical stimulation like physical exercise (Peng et al. 2022). Collectively, these findings underscore the multifaceted nature of RCN2 and its potential implications in diverse areas of biology and medicine.

Notably, while previous studies have primarily characterized RCN2 in the context of cancer, cardiovascular disease, and peripheral metabolic regulation, the present study extends these findings to the central nervous system and demonstrates that RCN2 modulates diabetes‐associated cognitive impairment through mechanisms involving neuroinflammation, synaptic regulation, and GSK3β–Tau signaling.

While RCN2 has been implicated in various diabetes‐related processes such as vascular calcification and atherosclerosis (Li et al. 2022; Chang et al. 2019), further investigation is needed to understand the mechanisms and potential therapeutic implications of RCN2 in diabetes. Notably, we showed that overload of glucose burden in the long‐term may exhaust the secretion of RCN2. Additionally, neuroinflammation was observed in HT22 with high glucose stimulation. This indicated that RCN2 appears to be involved in neurological diseases associated with inflammation, which is the hallmark of most nerve diseases. In this study, we showed that RCN2 knockdown in HT22 cells leads to altered synapsin expression. Mouse models demonstrated that knockout RCN2 in the hippocampus presented worse performance, while overexpression of RCN2 in hippocampus exhibited better performance during behavior experiments, verifying our in vitro results.

Tau protein and GSK3β are implicated as crucial factors in AD pathogenesis, with Tau undergoing hyperphosphorylation to form neurofibrillary tangles, a characteristic hallmark of AD (Wang et al. 2013; Lauretti et al. 2020). Inhibition of GSK3β has been shown to mitigate tau phosphorylation and improve cognitive deficits in AD models (Sayas and Avila 2021; Sen et al. 2007). Our results demonstrated that RCN2 deficiency in HT22 cells led to tau downregulation and reduced GSK3β activity. These results indicate that RCN2 depletion may regulate Alzheimer's disease‐associated signaling cascades, and modulating RCN2 activity holds promise as a therapeutic target for AD intervention, as it may impact multiple key players in AD pathogenesis.

In conclusion, we propose that RCN2 may serve as a pathological mechanism for cognitive impairment in individuals with diabetes and could potentially be targeted for the treatment of diabetic dementia.

## 5 Limitations

Notably, several study limitations warrant consideration. First, while db/db mice are a widely used model for type 2 diabetes, the control group in this study consisted of C57BL/6J wild‐type littermates rather than db/m mice. Given the use of genetically modified mice (RCN2‐floxed and CamkII‐Cre) on the C57BL/6J background, we selected C57BL/6J wild‐type mice as controls to maintain consistency across all experimental groups. Second, the experiments were conducted on mouse models or in vitro, and further research is needed to determine if the same effects would be observed in humans. Additionally, the specific mechanisms through which RCN2 influences cognitive function and the potential side effects of manipulating RCN2 levels require further investigation.

## Author Contributions

G.‐Q. X. and X. C. designed and supervised the experiments, analyzed results, and co‐wrote the manuscript. T. Z. and S.‐M. T. carried out most of the experiments and drafted the manuscript. B. H., L.‐Q. X., and Y.‐L. C. helped to conduct animal experiments. J.‐H. C. helped to generate and analyze data.

## Ethics Statement

All animal care protocols and experiments were reviewed and approved by the Animal Care and Use Committees of the Laboratory Animal Research Center at Xiangya Medical School of Central South University.

## Conflicts of Interest

The authors declare no conflicts of interest.

## Data Availability

Research data are not shared.
